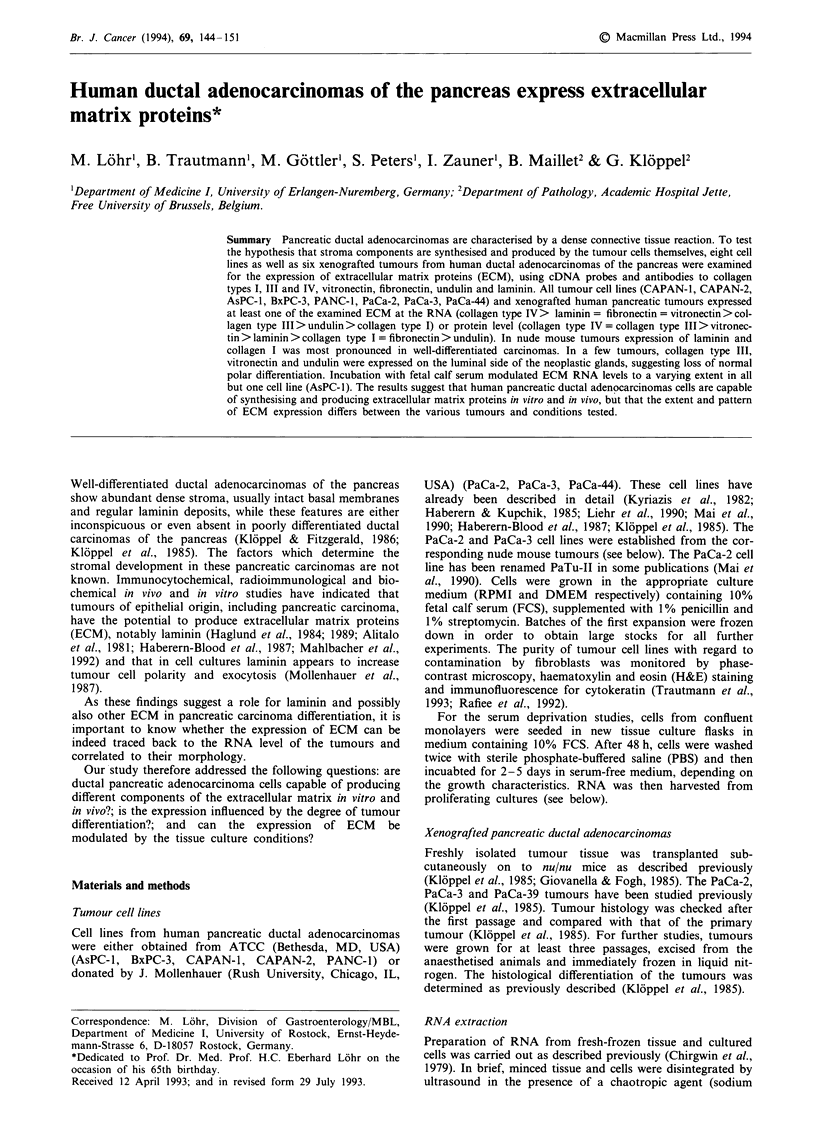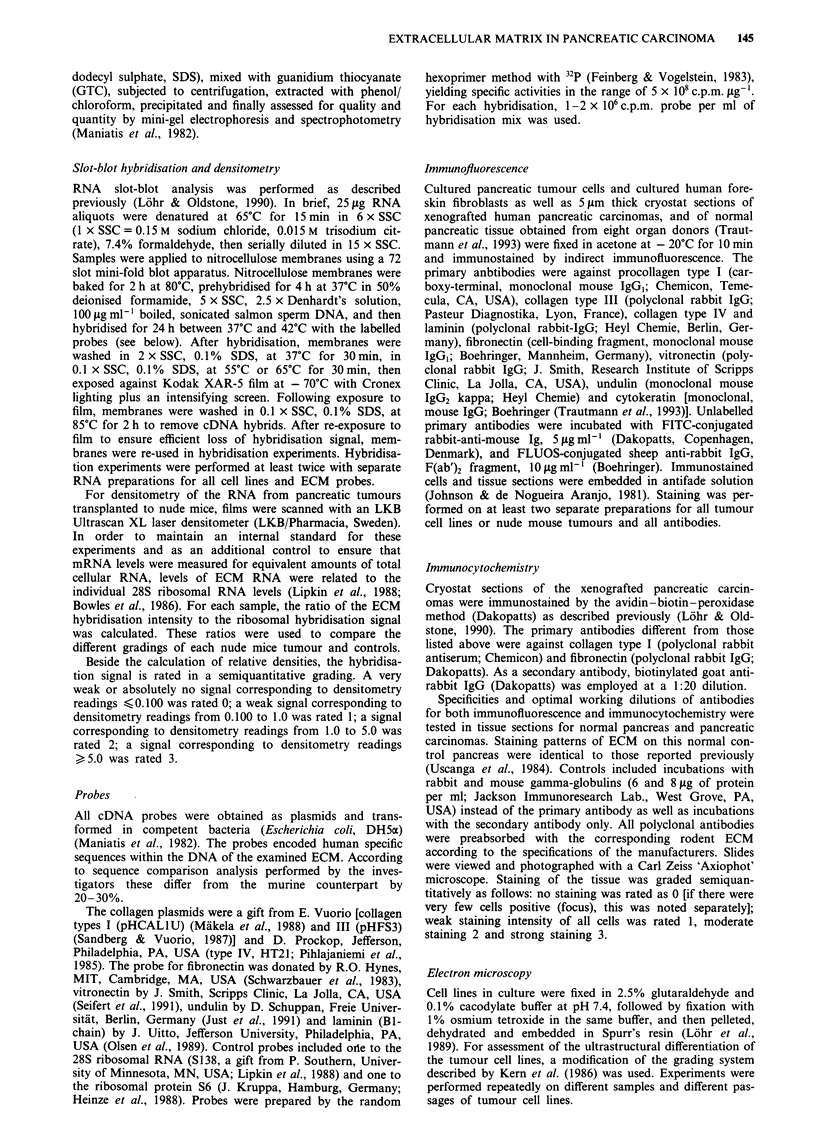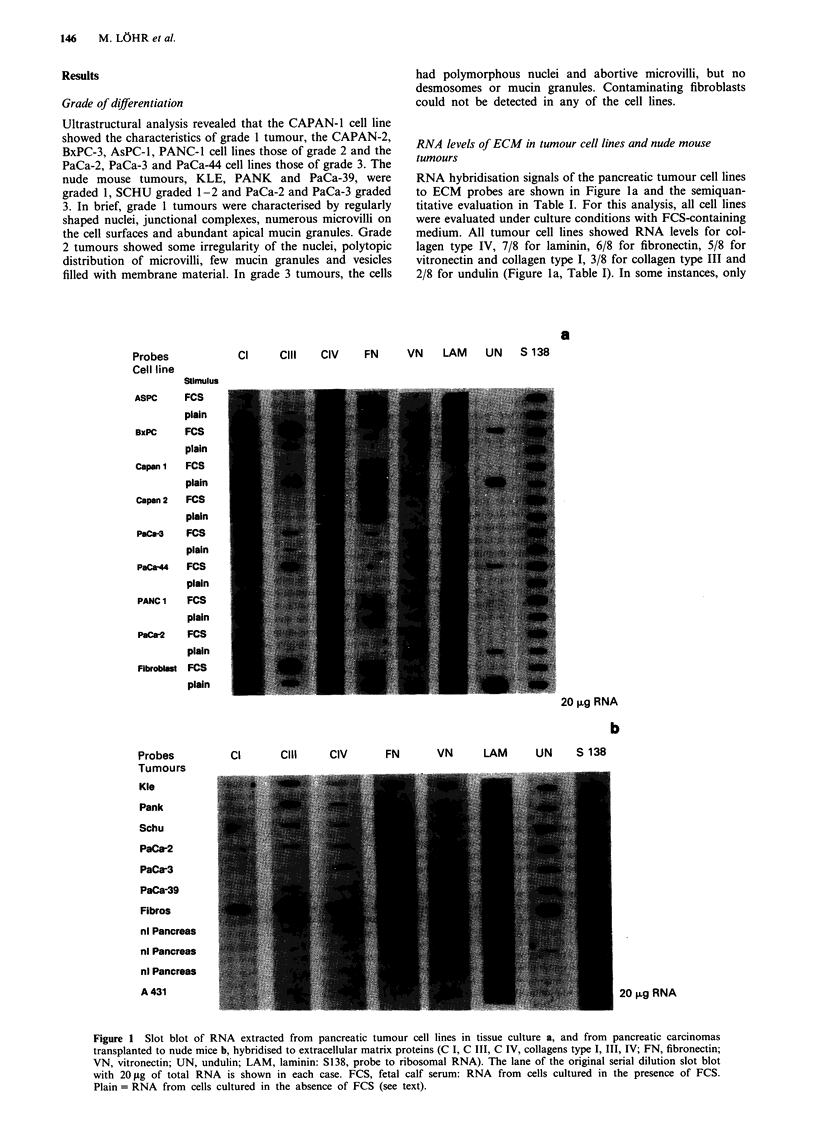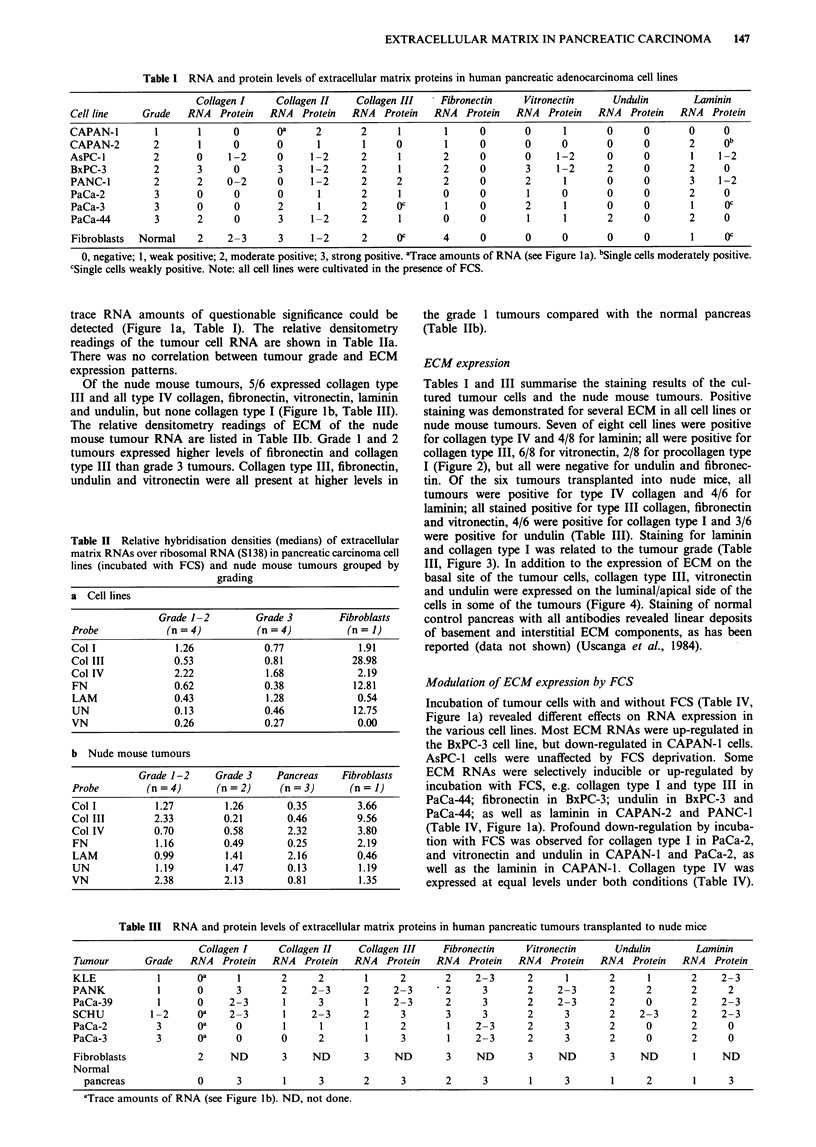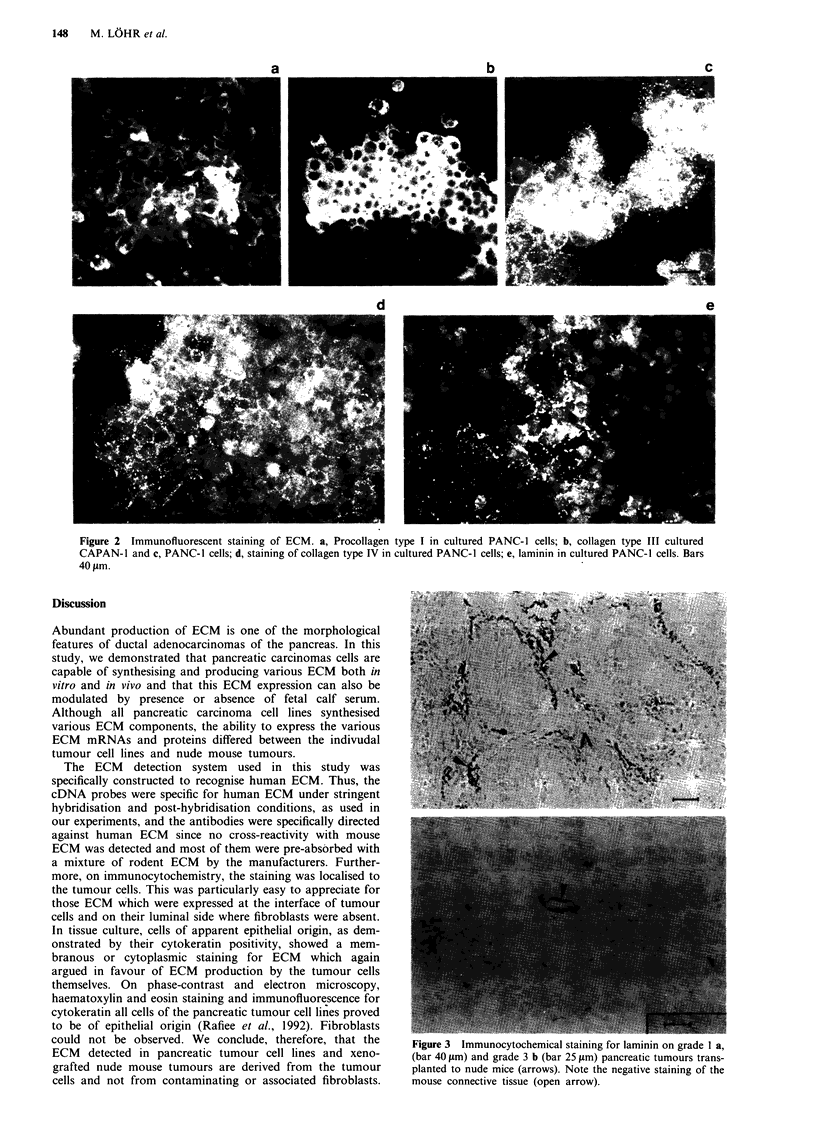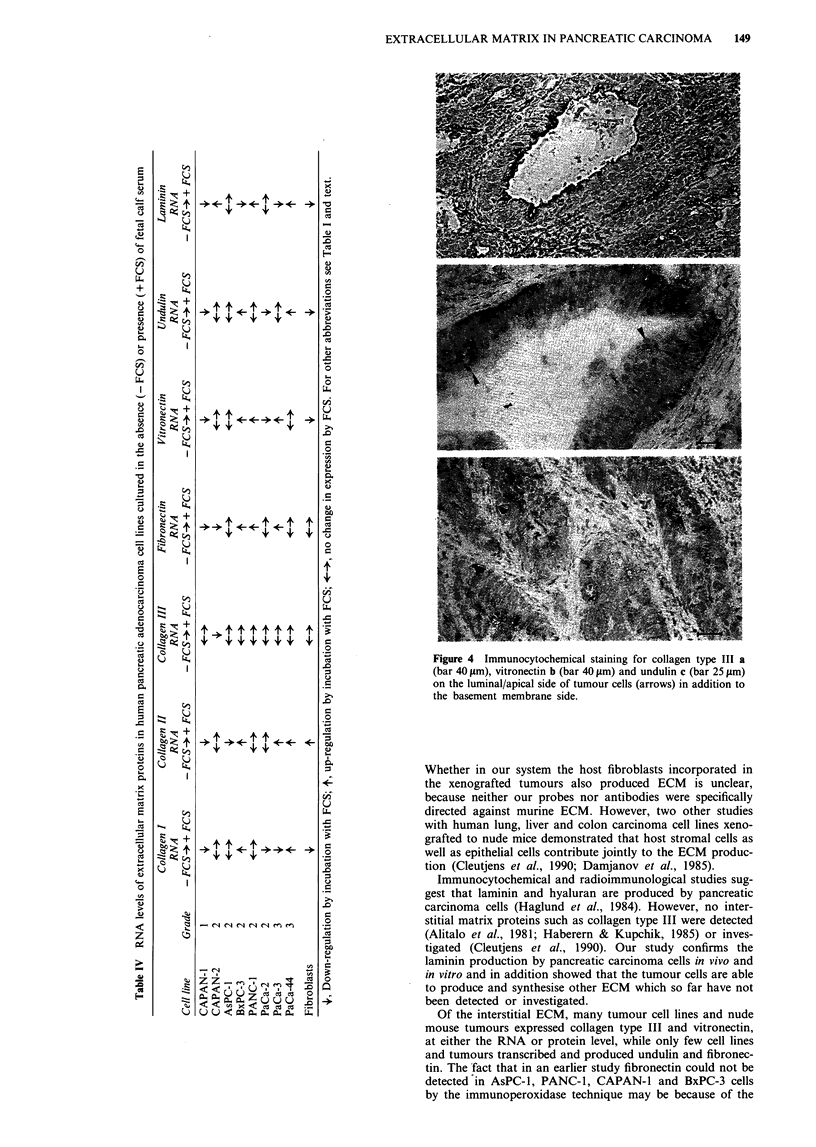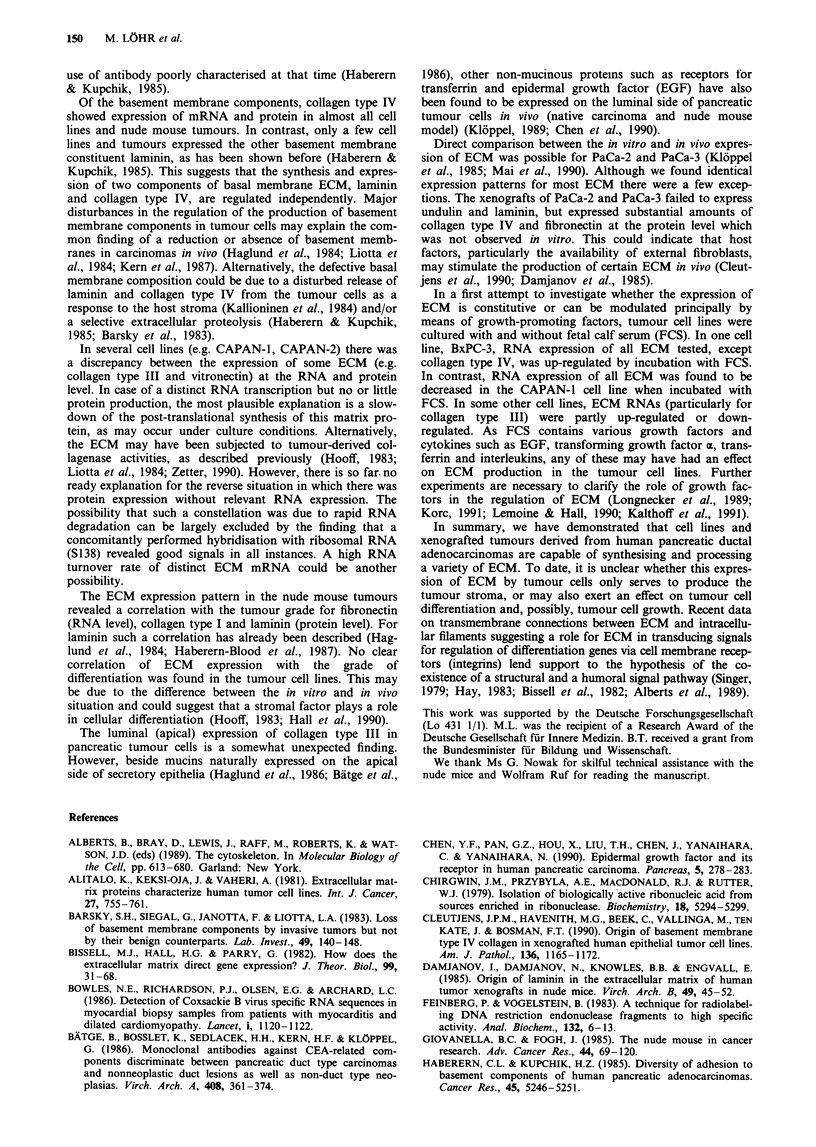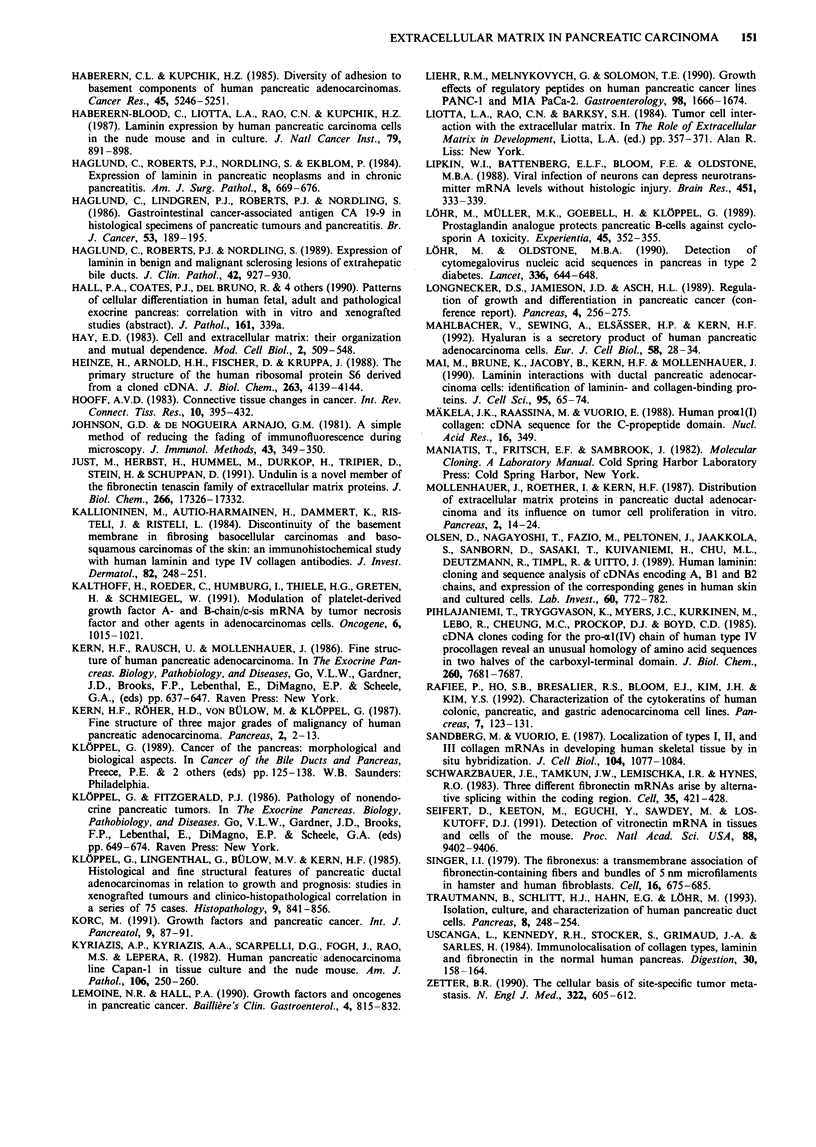# Human ductal adenocarcinomas of the pancreas express extracellular matrix proteins.

**DOI:** 10.1038/bjc.1994.24

**Published:** 1994-01

**Authors:** M. Löhr, B. Trautmann, M. Göttler, S. Peters, I. Zauner, B. Maillet, G. Klöppel

**Affiliations:** Department of Medicine I, University of Erlangen-Nuremberg, Germany.

## Abstract

**Images:**


Br. J. Cancer (1994), 69, 144-151 © Macmillan Press Ltd., 1994

### Human ductal adenocarcinomas of the pancreas express extracellular matrix proteins*

M. Löhr[1], B. Trautmann[1], M. Göttler[1], S. Peters[1], I. Zauner[1], B. Maillet[2] & G. Klöppel[2]

[1]Department of Medicine I, University of Erlangen-Nuremberg, Germany; [2]Department of Pathology, Academic Hospital Jette, Free University of Brussels, Belgium.

**Summary** Pancreatic ductal adenocarcinomas are characterised by a dense connective tissue reaction. To test the hypothesis that stroma components are synthesised and produced by the tumour cells themselves, eight cell lines as well as six xenografted tumours from human ductal adenocarcinomas of the pancreas were examined for the expression of extracellular matrix proteins (ECM), using cDNA probes and antibodies to collagen types I, III and IV, vitronectin, fibronectin, undulin and laminin. All tumour cell lines (CAPAN-1, CAPAN-2, AsPC-1, BxPC-3, PANC-1, PaCa-2, PaCa-3, PaCa-44) and xenografted human pancreatic tumours expressed at least one of the examined ECM at the RNA (collagen type IV > laminin = fibronectin = vitronectin > collagen type III > undulin > collagen type I) or protein level (collagen type IV = collagen type III > vitronectin > laminin > collagen type I = fibronectin > undulin). In nude mouse tumours expression of laminin and collagen I was most pronounced in well-differentiated carcinomas. In a few tumours, collagen type III, vitronectin and undulin were expressed on the luminal side of the neoplastic glands, suggesting loss of normal polar differentiation. Incubation with fetal calf serum modulated ECM RNA levels to a varying extent in all but one cell line (AsPC-1). The results suggest that human pancreatic ductal adenocarcinomas cells are capable of synthesising and producing extracellular matrix proteins *in vitro* and *in vivo*, but that the extent and pattern of ECM expression differs between the various tumours and conditions tested.

Well-differentiated ductal adenocarcinomas of the pancreas show abundant dense stroma, usually intact basal membranes and regular laminin deposits, while these features are either inconspicuous or even absent in poorly differentiated ductal carcinomas of the pancreas (Klöppel & Fitzgerald, 1986; Klöppel *et al*., 1985). The factors which determine the stromal development in these pancreatic carcinomas are not known. Immunocytochemical, radioimmunological and biochemical *in vivo* and *in vitro* studies have indicated that tumours of epithelial origin, including pancreatic carcinoma, have the potential to produce extracellular matrix proteins (ECM), notably laminin (Haglund *et al*., 1984; 1989; Alitalo *et al*., 1981; Haberern-Blood *et al*., 1987; Mahlbacher *et al*., 1992) and that in cell cultures laminin appears to increase tumour cell polarity and exocytosis (Mollenhauer *et al*., 1987).

As these findings suggest a role for laminin and possibly also other ECM in pancreatic carcinoma differentiation, it is important to know whether the expression of ECM can be indeed traced back to the RNA level of the tumours and correlated to their morphology.

Our study therefore addressed the following questions: are ductal pancreatic adenocarcinoma cells capable of producing different components of the extracellular matrix *in vitro* and *in vivo*?; is the expression influenced by the degree of tumour differentiation?; and can the expression of ECM be modulated by the tissue culture conditions?

### Materials and methods

### *Tumour cell lines*

Cell lines from human pancreatic ductal adenocarcinomas were either obtained from ATCC (Bethesda, MD, USA) (AsPC-1, BxPC-3, CAPAN-1, CAPAN-2, PANC-1) or donated by J. Mollenhauer (Rush University, Chicago, IL, USA) (PaCa-2, PaCa-3, PaCa-44). These cell lines have already been described in detail (Kyriazis *et al*., 1982; Haberern & Kupchik, 1985; Liehr *et al*., 1990; Mai *et al*., 1990; Haberern-Blood *et al*., 1987; Klöppel *et al*., 1985). The PaCa-2 and PaCa-3 cell lines were established from the corresponding nude mouse tumours (see below). The PaCa-2 cell line has been renamed PaTu-II in some publications (Mai *et al*., 1990). Cells were grown in the appropriate culture medium (RPMI and DMEM respectively) containing 10% fetal calf serum (FCS), supplemented with 1% penicillin and 1% streptomycin. Batches of the first expansion were frozen down in order to obtain large stocks for all further experiments. The purity of tumour cell lines with regard to contamination by fibroblasts was monitored by phase-contrast microscopy, haematoxylin and eosin (H&E) staining and immunofluorescence for cytokeratin (Trautmann *et al*., 1993; Rafiee *et al*., 1992).

For the serum deprivation studies, cells from confluent monolayers were seeded in new tissue culture flasks in medium containing 10% FCS. After 48 h, cells were washed twice with sterile phosphate-buffered saline (PBS) and then incuabted for 2-5 days in serum-free medium, depending on the growth characteristics. RNA was then harvested from proliferating cultures (see below).

### *Xenografted pancreatic ductal adenocarcinomas*

Freshly isolated tumour tissue was transplanted subcutaneously on to *nu/nu* mice as described previously (Klöppel *et al*., 1985; Giovanella & Fogh, 1985). The PaCa-2, PaCa-3 and PaCa-39 tumours have been studied previously (Klöppel *et al*., 1985). Tumour histology was checked after the first passage and compared with that of the primary tumour (Klöppel *et al*., 1985). For further studies, tumours were grown for at least three passages, excised from the anaesthetised animals and immediately frozen in liquid nitrogen. The histological differentiation of the tumours was determined as previously described (Klöppel *et al*., 1985).

### *RNA extraction*

Preparation of RNA from fresh-frozen tissue and cultured cells was carried out as described previously (Chirgwin *et al*., 1979). In brief, minced tissue and cells were disintegrated by ultrasound in the presence of a chaotropic agent (sodium

Correspondence: M. Löhr, Division of Gastroenterology/MBL, Department of Medicine I, University of Rostock, Ernst-Heydemann-Strasse 6, D-18057 Rostock, Germany.
*Dedicated to Prof. Dr. Med. Prof. H.C. Eberhard Löhr on the occasion of his 65th birthday.
Received 12 April 1993; and in revised form 29 July 1993.

dodecyl sulphate, SDS), mixed with guanidium thiocyanate (GTC), subjected to centrifugation, extracted with phenol/chloroform, precipitated and finally assessed for quality and quantity by mini-gel electrophoresis and spectrophotometry (Maniatis *et al.*, 1982).

*Slot-blot hybridisation and densitometry*

RNA slot-blot analysis was performed as described previously (Löhr & Oldstone, 1990). In brief, 25 μg RNA aliquots were denatured at 65°C for 15 min in 6 × SSC (1 × SSC = 0.15 M sodium chloride, 0.015 M trisodium citrate), 7.4% formaldehyde, then serially diluted in 15 × SSC. Samples were applied to nitrocellulose membranes using a 72 slot mini-fold blot apparatus. Nitrocellulose membranes were baked for 2 h at 80°C, prehybridised for 4 h at 37°C in 50% deionised formamide, 5 × SSC, 2.5 × Denhardt's solution, 100 $\mu g\ ml^{-1}$ boiled, sonicated salmon sperm DNA, and then hybridised for 24 h between 37°C and 42°C with the labelled probes (see below). After hybridisation, membranes were washed in 2 × SSC, 0.1% SDS, at 37°C for 30 min, in 0.1 × SSC, 0.1% SDS, at 55°C or 65°C for 30 min, then exposed against Kodak XAR-5 film at − 70°C with Cronex lighting plus an intensifying screen. Following exposure to film, membranes were washed in 0.1 × SSC, 0.1% SDS, at 85°C for 2 h to remove cDNA hybrids. After re-exposure to film to ensure efficient loss of hybridisation signal, membranes were re-used in hybridisation experiments. Hybridisation experiments were performed at least twice with separate RNA preparations for all cell lines and ECM probes.

For densitometry of the RNA from pancreatic tumours transplanted to nude mice, films were scanned with an LKB Ultrascan XL laser densitometer (LKB/Pharmacia, Sweden). In order to maintain an internal standard for these experiments and as an additional control to ensure that mRNA levels were measured for equivalent amounts of total cellular RNA, levels of ECM RNA were related to the individual 28S ribosomal RNA levels (Lipkin *et al.*, 1988; Bowles *et al.*, 1986). For each sample, the ratio of the ECM hybridisation intensity to the ribosomal hybridisation signal was calculated. These ratios were used to compare the different gradings of each nude mice tumour and controls.

Beside the calculation of relative densities, the hybridisation signal is rated in a semiquantitative grading. A very weak or absolutely no signal corresponding to densitometry readings $\leqslant 0.100$ was rated 0; a weak signal corresponding to densitometry readings from 0.100 to 1.0 was rated 1; a signal corresponding to densitometry readings from 1.0 to 5.0 was rated 2; a signal corresponding to densitometry readings $\geqslant 5.0$ was rated 3.

*Probes*

All cDNA probes were obtained as plasmids and transformed in competent bacteria (*Escherichia coli*, DH5α) (Maniatis *et al.*, 1982). The probes encoded human specific sequences within the DNA of the examined ECM. According to sequence comparison analysis performed by the investigators these differ from the murine counterpart by 20–30%.

The collagen plasmids were a gift from E. Vuorio [collagen types I (pHCAL1U) (Mäkela *et al.*, 1988) and III (pHFS3) (Sandberg & Vuorio, 1987)] and D. Prockop, Jefferson, Philadelphia, PA, USA (type IV, HT21; Pihlajaniemi *et al.*, 1985). The probe for fibronectin was donated by R.O. Hynes, MIT, Cambridge, MA, USA (Schwarzbauer *et al.*, 1983), vitronectin by J. Smith, Scripps Clinic, La Jolla, CA, USA (Seifert *et al.*, 1991), undulin by D. Schuppan, Freie Universität, Berlin, Germany (Just *et al.*, 1991) and laminin (B1-chain) by J. Uitto, Jefferson University, Philadelphia, PA, USA (Olsen *et al.*, 1989). Control probes included one to the 28S ribosomal RNA (S138, a gift from P. Southern, University of Minnesota, MN, USA; Lipkin *et al.*, 1988) and one to the ribosomal protein S6 (J. Kruppa, Hamburg, Germany; Heinze *et al.*, 1988). Probes were prepared by the random hexoprimer method with $^{32}P$ (Feinberg & Vogelstein, 1983), yielding specific activities in the range of $5 \times 10^8$ c.p.m. $\mu g^{-1}$. For each hybridisation, $1-2 \times 10^6$ c.p.m. probe per ml of hybridisation mix was used.

*Immunofluorescence*

Cultured pancreatic tumour cells and cultured human foreskin fibroblasts as well as 5 μm thick cryostat sections of xenografted human pancreatic carcinomas, and of normal pancreatic tissue obtained from eight organ donors (Trautmann *et al.*, 1993) were fixed in acetone at − 20°C for 10 min and immunostained by indirect immunofluorescence. The primary anbtibodies were against procollagen type I (carboxy-terminal, monoclonal mouse $IgG_1$; Chemicon, Temecula, CA, USA), collagen type III (polyclonal rabbit IgG; Pasteur Diagnostika, Lyon, France), collagen type IV and laminin (polyclonal rabbit-IgG; Heyl Chemie, Berlin, Germany), fibronectin (cell-binding fragment, monoclonal mouse $IgG_1$; Boehringer, Mannheim, Germany), vitronectin (polyclonal rabbit IgG; J. Smith, Research Institute of Scripps Clinic, La Jolla, CA, USA), undulin (monoclonal mouse $IgG_2$ kappa; Heyl Chemie) and cytokeratin [monoclonal, mouse IgG; Boehringer (Trautmann *et al.*, 1993)]. Unlabelled primary antibodies were incubated with FITC-conjugated rabbit-anti-mouse Ig, 5 $\mu g\ ml^{-1}$ (Dakopatts, Copenhagen, Denmark), and FLUOS-conjugated sheep anti-rabbit IgG, $F(ab')_2$ fragment, 10 $\mu g\ ml^{-1}$ (Boehringer). Immunostained cells and tissue sections were embedded in antifade solution (Johnson & de Nogueira Aranjo, 1981). Staining was performed on at least two separate preparations for all tumour cell lines or nude mouse tumours and all antibodies.

*Immunocytochemistry*

Cryostat sections of the xenografted pancreatic carcinomas were immunostained by the avidin–biotin–peroxidase method (Dakopatts) as described previously (Löhr & Oldstone, 1990). The primary antibodies different from those listed above were against collagen type I (polyclonal rabbit antiserum; Chemicon) and fibronectin (polyclonal rabbit IgG; Dakopatts). As a secondary antibody, biotinylated goat anti-rabbit IgG (Dakopatts) was employed at a 1:20 dilution.

Specificities and optimal working dilutions of antibodies for both immunofluorescence and immunocytochemistry were tested in tissue sections for normal pancreas and pancreatic carcinomas. Staining patterns of ECM on this normal control pancreas were identical to those reported previously (Uscanga *et al.*, 1984). Controls included incubations with rabbit and mouse gamma-globulins (6 and 8 μg of protein per ml; Jackson Immunoresearch Lab., West Grove, PA, USA) instead of the primary antibody as well as incubations with the secondary antibody only. All polyclonal antibodies were preabsorbed with the corresponding rodent ECM according to the specifications of the manufacturers. Slides were viewed and photographed with a Carl Zeiss 'Axiophot' microscope. Staining of the tissue was graded semiquantitatively as follows: no staining was rated as 0 [if there were very few cells positive (focus), this was noted separately]; weak staining intensity of all cells was rated 1, moderate staining 2 and strong staining 3.

*Electron microscopy*

Cell lines in culture were fixed in 2.5% glutaraldehyde and 0.1% cacodylate buffer at pH 7.4, followed by fixation with 1% osmium tetroxide in the same buffer, and then pelleted, dehydrated and embedded in Spurr's resin (Löhr *et al.*, 1989). For assessment of the ultrastructural differentiation of the tumour cell lines, a modification of the grading system described by Kern *et al.* (1986) was used. Experiments were performed repeatedly on different samples and different passages of tumour cell lines.

### Results

### *Grade of differentiation*

Ultrastructural analysis revealed that the CAPAN-1 cell line showed the characteristics of grade 1 tumour, the CAPAN-2, BxPC-3, AsPC-1, PANC-1 cell lines those of grade 2 and the PaCa-2, PaCa-3 and PaCa-44 cell lines those of grade 3. The nude mouse tumours, KLE, PANK and PaCa-39, were graded 1, SCHU graded 1–2 and PaCa-2 and PaCa-3 graded 3. In brief, grade 1 tumours were characterised by regularly shaped nuclei, junctional complexes, numerous microvilli on the cell surfaces and abundant apical mucin granules. Grade 2 tumours showed some irregularity of the nuclei, polytopic distribution of microvilli, few mucin granules and vesicles filled with membrane material. In grade 3 tumours, the cells had polymorphous nuclei and abortive microvilli, but no desmosomes or mucin granules. Contaminating fibroblasts could not be detected in any of the cell lines.

### *RNA levels of ECM in tumour cell lines and nude mouse tumours*

RNA hybridisation signals of the pancreatic tumour cell lines to ECM probes are shown in Figure 1a and the semiquantitative evaluation in Table I. For this analysis, all cell lines were evaluated under culture conditions with FCS-containing medium. All tumour cell lines showed RNA levels for collagen type IV, 7/8 for laminin, 6/8 for fibronectin, 5/8 for vitronectin and collagen type I, 3/8 for collagen type III and 2/8 for undulin (Figure 1a, Table I). In some instances, only

**Figure 1** Slot blot of RNA extracted from pancreatic tumour cell lines in tissue culture **a**, and from pancreatic carcinomas transplanted to nude mice **b**, hybridised to extracellular matrix proteins (C I, C III, C IV, collagens type I, III, IV; FN, fibronectin; VN, vitronectin; UN, undulin; LAM, laminin: S138, probe to ribosomal RNA). The lane of the original serial dilution slot blot with 20 μg of total RNA is shown in each case. FCS, fetal calf serum: RNA from cells cultured in the presence of FCS. Plain = RNA from cells cultured in the absence of FCS (see text).

**Table I** RNA and protein levels of extracellular matrix proteins in human pancreatic adenocarcinoma cell lines

| | | *Collagen I* | | *Collagen II* | | *Collagen III* | | *Fibronectin* | | *Vitronectin* | | *Undulin* | | *Laminin* | |
|---|---|---|---|---|---|---|---|---|---|---|---|---|---|---|---|
| *Cell line* | *Grade* | *RNA* | *Protein* | *RNA* | *Protein* | *RNA* | *Protein* | *RNA* | *Protein* | *RNA* | *Protein* | *RNA* | *Protein* | *RNA* | *Protein* |
| CAPAN-1 | 1 | 1 | 0 | 0[a] | 2 | 2 | 1 | 1 | 0 | 0 | 1 | 0 | 0 | 0 | 0 |
| CAPAN-2 | 2 | 1 | 0 | 0 | 1 | 1 | 0 | 1 | 0 | 0 | 0 | 0 | 0 | 2 | 0[b] |
| AsPC-1 | 2 | 0 | 1–2 | 0 | 1–2 | 2 | 1 | 2 | 0 | 0 | 1–2 | 0 | 0 | 1 | 1–2 |
| BxPC-3 | 2 | 3 | 0 | 3 | 1–2 | 2 | 1 | 2 | 0 | 3 | 1–2 | 2 | 0 | 2 | 0 |
| PANC-1 | 2 | 2 | 0–2 | 0 | 1–2 | 2 | 2 | 2 | 0 | 2 | 1 | 0 | 0 | 3 | 1–2 |
| PaCa-2 | 3 | 0 | 0 | 0 | 1 | 2 | 1 | 0 | 0 | 1 | 0 | 0 | 0 | 2 | 0 |
| PaCa-3 | 3 | 0 | 0 | 2 | 1 | 2 | 0[c] | 1 | 0 | 2 | 1 | 0 | 0 | 1 | 0[c] |
| PaCa-44 | 3 | 2 | 0 | 3 | 1–2 | 2 | 1 | 0 | 0 | 1 | 1 | 2 | 0 | 2 | 0 |
| Fibroblasts | Normal | 2 | 2–3 | 3 | 1–2 | 2 | 0[c] | 4 | 0 | 0 | 0 | 0 | 0 | 1 | 0[c] |

0, negative; 1, weak positive; 2, moderate positive; 3, strong positive. [a]Trace amounts of RNA (see Figure 1a). [b]Single cells moderately positive. [c]Single cells weakly positive. Note: all cell lines were cultivated in the presence of FCS.

trace RNA amounts of questionable significance could be detected (Figure 1a, Table I). The relative densitometry readings of the tumour cell RNA are shown in Table IIa. There was no correlation between tumour grade and ECM expression patterns.

Of the nude mouse tumours, 5/6 expressed collagen type III and all type IV collagen, fibronectin, vitronectin, laminin and undulin, but none collagen type I (Figure 1b, Table III). The relative densitometry readings of ECM of the nude mouse tumour RNA are listed in Table IIb. Grade 1 and 2 tumours expressed higher levels of fibronectin and collagen type III than grade 3 tumours. Collagen type III, fibronectin, undulin and vitronectin were all present at higher levels in the grade 1 tumours compared with the normal pancreas (Table IIb).

**Table II** Relative hybridisation densities (medians) of extracellular matrix RNAs over ribosomal RNA (S138) in pancreatic carcinoma cell lines (incubated with FCS) and nude mouse tumours grouped by grading

**a** Cell lines

| *Probe* | *Grade 1–2* (n = *4*) | *Grade 3* (n = *4*) | *Fibroblasts* (n = *1*) |
|---|---|---|---|
| Col I | 1.26 | 0.77 | 1.91 |
| Col III | 0.53 | 0.81 | 28.98 |
| Col IV | 2.22 | 1.68 | 2.19 |
| FN | 0.62 | 0.38 | 12.81 |
| LAM | 0.43 | 1.28 | 0.54 |
| UN | 0.13 | 0.46 | 12.75 |
| VN | 0.26 | 0.27 | 0.00 |

**b** Nude mouse tumours

| *Probe* | *Grade 1–2* (n = *4*) | *Grade 3* (n = *2*) | *Pancreas* (n = *3*) | *Fibroblasts* (n = *1*) |
|---|---|---|---|---|
| Col I | 1.27 | 1.26 | 0.35 | 3.66 |
| Col III | 2.33 | 0.21 | 0.46 | 9.56 |
| Col IV | 0.70 | 0.58 | 2.32 | 3.80 |
| FN | 1.16 | 0.49 | 0.25 | 2.19 |
| LAM | 0.99 | 1.41 | 2.16 | 0.46 |
| UN | 1.19 | 1.47 | 0.13 | 1.19 |
| VN | 2.38 | 2.13 | 0.81 | 1.35 |

*ECM expression*

Tables I and III summarise the staining results of the cultured tumour cells and the nude mouse tumours. Positive staining was demonstrated for several ECM in all cell lines or nude mouse tumours. Seven of eight cell lines were positive for collagen type IV and 4/8 for laminin; all were positive for collagen type III, 6/8 for vitronectin, 2/8 for procollagen type I (Figure 2), but all were negative for undulin and fibronectin. Of the six tumours transplanted into nude mice, all tumours were positive for type IV collagen and 4/6 for laminin; all stained positive for type III collagen, fibronectin and vitronectin, 4/6 were positive for collagen type I and 3/6 were positive for undulin (Table III). Staining for laminin and collagen type I was related to the tumour grade (Table III, Figure 3). In addition to the expression of ECM on the basal site of the tumour cells, collagen type III, vitronectin and undulin were expressed on the luminal/apical side of the cells in some of the tumours (Figure 4). Staining of normal control pancreas with all antibodies revealed linear deposits of basement and interstitial ECM components, as has been reported (data not shown) (Uscanga *et al.*, 1984).

*Modulation of ECM expression by FCS*

Incubation of tumour cells with and without FCS (Table IV, Figure 1a) revealed different effects on RNA expression in the various cell lines. Most ECM RNAs were up-regulated in the BxPC-3 cell line, but down-regulated in CAPAN-1 cells. AsPC-1 cells were unaffected by FCS deprivation. Some ECM RNAs were selectively inducible or up-regulated by incubation with FCS, e.g. collagen type I and type III in PaCa-44; fibronectin in BxPC-3; undulin in BxPC-3 and PaCa-44; as well as laminin in CAPAN-2 and PANC-1 (Table IV, Figure 1a). Profound down-regulation by incubation with FCS was observed for collagen type I in PaCa-2, and vitronectin and undulin in CAPAN-1 and PaCa-2, as well as the laminin in CAPAN-1. Collagen type IV was expressed at equal levels under both conditions (Table IV).

**Table III** RNA and protein levels of extracellular matrix proteins in human pancreatic tumours transplanted to nude mice

| | | *Collagen I* | | *Collagen II* | | *Collagen III* | | *Fibronectin* | | *Vitronectin* | | *Undulin* | | *Laminin* | |
|---|---|---|---|---|---|---|---|---|---|---|---|---|---|---|---|
| *Tumour* | *Grade* | *RNA* | *Protein* | *RNA* | *Protein* | *RNA* | *Protein* | *RNA* | *Protein* | *RNA* | *Protein* | *RNA* | *Protein* | *RNA* | *Protein* |
| KLE | 1 | 0[a] | 1 | 2 | 2 | 1 | 2 | 2 | 2–3 | 2 | 1 | 2 | 1 | 2 | 2–3 |
| PANK | 1 | 0 | 3 | 2 | 2–3 | 2 | 2–3 | 2 | 3 | 2 | 2–3 | 2 | 2 | 2 | 2 |
| PaCa-39 | 1 | 0 | 2–3 | 1 | 3 | 1 | 2–3 | 2 | 3 | 2 | 2–3 | 2 | 0 | 2 | 2–3 |
| SCHU | 1–2 | 0[a] | 2–3 | 1 | 2–3 | 2 | 3 | 3 | 3 | 2 | 3 | 2 | 2–3 | 2 | 2–3 |
| PaCa-2 | 3 | 0[a] | 0 | 1 | 1 | 1 | 2 | 1 | 2–3 | 2 | 3 | 2 | 0 | 2 | 0 |
| PaCa-3 | 3 | 0[a] | 0 | 0 | 2 | 1 | 3 | 1 | 2–3 | 2 | 3 | 2 | 0 | 2 | 0 |
| Fibroblasts | | 2 | ND | 3 | ND | 3 | ND | 3 | ND | 3 | ND | 3 | ND | 1 | ND |
| Normal pancreas | | 0 | 3 | 1 | 3 | 2 | 3 | 2 | 3 | 1 | 3 | 1 | 2 | 1 | 3 |

[a]Trace amounts of RNA (see Figure 1b). ND, not done.

**Figure 2** Immunofluorescent staining of ECM. **a**, Procollagen type I in cultured PANC-1 cells; **b**, collagen type III cultured CAPAN-1 and **c**, PANC-1 cells; **d**, staining of collagen type IV in cultured PANC-1 cells; **e**, laminin in cultured PANC-1 cells. Bars 40 μm.

### Discussion

Abundant production of ECM is one of the morphological features of ductal adenocarcinomas of the pancreas. In this study, we demonstrated that pancreatic carcinomas cells are capable of synthesising and producing various ECM both *in vitro* and *in vivo* and that this ECM expression can also be modulated by presence or absence of fetal calf serum. Although all pancreatic carcinoma cell lines synthesised various ECM components, the ability to express the various ECM mRNAs and proteins differed between the indivudal tumour cell lines and nude mouse tumours.

The ECM detection system used in this study was specifically constructed to recognise human ECM. Thus, the cDNA probes were specific for human ECM under stringent hybridisation and post-hybridisation conditions, as used in our experiments, and the antibodies were specifically directed against human ECM since no cross-reactivity with mouse ECM was detected and most of them were pre-absorbed with a mixture of rodent ECM by the manufacturers. Furthermore, on immunocytochemistry, the staining was localised to the tumour cells. This was particularly easy to appreciate for those ECM which were expressed at the interface of tumour cells and on their luminal side where fibroblasts were absent. In tissue culture, cells of apparent epithelial origin, as demonstrated by their cytokeratin positivity, showed a membranous or cytoplasmic staining for ECM which again argued in favour of ECM production by the tumour cells themselves. On phase-contrast and electron microscopy, haematoxylin and eosin staining and immunofluorescence for cytokeratin all cells of the pancreatic tumour cell lines proved to be of epithelial origin (Rafiee *et al.*, 1992). Fibroblasts could not be observed. We conclude, therefore, that the ECM detected in pancreatic tumour cell lines and xenografted nude mouse tumours are derived from the tumour cells and not from contaminating or associated fibroblasts.

**Figure 3** Immunocytochemical staining for laminin on grade 1 **a**, (bar 40 μm) and grade 3 **b** (bar 25 μm) pancreatic tumours transplanted to nude mice (arrows). Note the negative staining of the mouse connective tissue (open arrow).

**Table IV** RNA levels of extracellular matrix proteins in human pancreatic adenocarcinoma cell lines cultured in the absence (−FCS) or presence (+FCS) of fetal calf serum

| *Cell line* | *Grade* | *Collagen I RNA −FCS→+FCS* | *Collagen II RNA −FCS→+FCS* | *Collagen III RNA −FCS→+FCS* | *Fibronectin RNA −FCS→+FCS* | *Vitronectin RNA −FCS→+FCS* | *Undulin RNA −FCS→+FCS* | *Laminin RNA −FCS→+FCS* |
|---|---|---|---|---|---|---|---|---|
| CAPAN-1 | 1 | ↓ | ↓ | ↔ | ↓ | ↓ | ↓ | ↓ |
| CAPAN-2 | 2 | ↔ | ↔ | ↓ | ↓ | ↔ | ↔ | ↑ |
| AsPC-1 | 2 | ↔ | ↓ | ↔ | ↔ | ↔ | ↔ | ↔ |
| BxPC-3 | 2 | ↑ | ↑ | ↔ | ↑ | ↑ | ↑ | ↓ |
| PANC-1 | 2 | ↔ | ↔ | ↔ | ↑ | ↑ | ↔ | ↑ |
| PaCa-2 | 2 | ↓ | ↔ | ↔ | ↔ | ↓ | ↓ | ↔ |
| PaCa-3 | 3 | ↓ | ↑ | ↔ | ↑ | ↑ | ↔ | ↓ |
| PaCa-44 | 3 | ↑ | ↑ | ↔ | ↔ | ↔ | ↑ | ↑ |
| Fibroblasts | | ↓ | ↑ | ↔ | ↔ | ↓ | ↓ | ↓ |

↓, Down-regulation by incubation with FCS; ↑, up-regulation by incubation with FCS; ↔, no change in expression by FCS. For other abbreviations see Table I and text.

**Figure 4** Immunocytochemical staining for collagen type III **a** (bar 40 μm), vitronectin **b** (bar 40 μm) and undulin **c** (bar 25 μm) on the luminal/apical side of tumour cells (arrows) in addition to the basement membrane side.

Whether in our system the host fibroblasts incorporated in the xenografted tumours also produced ECM is unclear, because neither our probes nor antibodies were specifically directed against murine ECM. However, two other studies with human lung, liver and colon carcinoma cell lines xenografted to nude mice demonstrated that host stromal cells as well as epithelial cells contribute jointly to the ECM production (Cleutjens *et al.*, 1990; Damjanov *et al.*, 1985).

Immunocytochemical and radioimmunological studies suggest that laminin and hyaluran are produced by pancreatic carcinoma cells (Haglund *et al.*, 1984). However, no interstitial matrix proteins such as collagen type III were detected (Alitalo *et al.*, 1981; Haberern & Kupchik, 1985) or investigated (Cleutjens *et al.*, 1990). Our study confirms the laminin production by pancreatic carcinoma cells *in vivo* and *in vitro* and in addition showed that the tumour cells are able to produce and synthesise other ECM which so far have not been detected or investigated.

Of the interstitial ECM, many tumour cell lines and nude mouse tumours expressed collagen type III and vitronectin, at either the RNA or protein level, while only few cell lines and tumours transcribed and produced undulin and fibronectin. The fact that in an earlier study fibronectin could not be detected in AsPC-1, PANC-1, CAPAN-1 and BxPC-3 cells by the immunoperoxidase technique may be because of the use of antibody poorly characterised at that time (Haberern & Kupchik, 1985).

Of the basement membrane components, collagen type IV showed expression of mRNA and protein in almost all cell lines and nude mouse tumours. In contrast, only a few cell lines and tumours expressed the other basement membrane constituent laminin, as has been shown before (Haberern & Kupchik, 1985). This suggests that the synthesis and expression of two components of basal membrane ECM, laminin and collagen type IV, are regulated independently. Major disturbances in the regulation of the production of basement membrane components in tumour cells may explain the common finding of a reduction or absence of basement membranes in carcinomas *in vivo* (Haglund *et al.*, 1984; Liotta *et al.*, 1984; Kern *et al.*, 1987). Alternatively, the defective basal membrane composition could be due to a disturbed release of laminin and collagen type IV from the tumour cells as a response to the host stroma (Kallioninen *et al.*, 1984) and/or a selective extracellular proteolysis (Haberern & Kupchik, 1985; Barsky *et al.*, 1983).

In several cell lines (e.g. CAPAN-1, CAPAN-2) there was a discrepancy between the expression of some ECM (e.g. collagen type III and vitronectin) at the RNA and protein level. In case of a distinct RNA transcription but no or little protein production, the most plausible explanation is a slowdown of the post-translational synthesis of this matrix protein, as may occur under culture conditions. Alternatively, the ECM may have been subjected to tumour-derived collagenase activities, as described previously (Hooff, 1983; Liotta *et al.*, 1984; Zetter, 1990). However, there is so far no ready explanation for the reverse situation in which there was protein expression without relevant RNA expression. The possibility that such a constellation was due to rapid RNA degradation can be largely excluded by the finding that a concomitantly performed hybridisation with ribosomal RNA (S138) revealed good signals in all instances. A high RNA turnover rate of distinct ECM mRNA could be another possibility.

The ECM expression pattern in the nude mouse tumours revealed a correlation with the tumour grade for fibronectin (RNA level), collagen type I and laminin (protein level). For laminin such a correlation has already been described (Haglund *et al.*, 1984; Haberern-Blood *et al.*, 1987). No clear correlation of ECM expression with the grade of differentiation was found in the tumour cell lines. This may be due to the difference between the *in vitro* and *in vivo* situation and could suggest that a stromal factor plays a role in cellular differentiation (Hooff, 1983; Hall *et al.*, 1990).

The luminal (apical) expression of collagen type III in pancreatic tumour cells is a somewhat unexpected finding. However, beside mucins naturally expressed on the apical side of secretory epithelia (Haglund *et al.*, 1986; Bätge *et al.*, 1986), other non-mucinous proteins such as receptors for transferrin and epidermal growth factor (EGF) have also been found to be expressed on the luminal side of pancreatic tumour cells *in vivo* (native carcinoma and nude mouse model) (Klöppel, 1989; Chen *et al.*, 1990).

Direct comparison between the *in vitro* and *in vivo* expression of ECM was possible for PaCa-2 and PaCa-3 (Klöppel *et al.*, 1985; Mai *et al.*, 1990). Although we found identical expression patterns for most ECM there were a few exceptions. The xenografts of PaCa-2 and PaCa-3 failed to express undulin and laminin, but expressed substantial amounts of collagen type IV and fibronectin at the protein level which was not observed *in vitro*. This could indicate that host factors, particularly the availability of external fibroblasts, may stimulate the production of certain ECM *in vivo* (Cleutjens *et al.*, 1990; Damjanov *et al.*, 1985).

In a first attempt to investigate whether the expression of ECM is constitutive or can be modulated principally by means of growth-promoting factors, tumour cell lines were cultured with and without fetal calf serum (FCS). In one cell line, BxPC-3, RNA expression of all ECM tested, except collagen type IV, was up-regulated by incubation with FCS. In contrast, RNA expression of all ECM was found to be decreased in the CAPAN-1 cell line when incubated with FCS. In some other cell lines, ECM RNAs (particularly for collagen type III) were partly up-regulated or down-regulated. As FCS contains various growth factors and cytokines such as EGF, transforming growth factor $\alpha$, transferrin and interleukins, any of these may have had an effect on ECM production in the tumour cell lines. Further experiments are necessary to clarify the role of growth factors in the regulation of ECM (Longnecker *et al.*, 1989; Korc, 1991; Lemoine & Hall, 1990; Kalthoff *et al.*, 1991).

In summary, we have demonstrated that cell lines and xenografted tumours derived from human pancreatic ductal adenocarcinomas are capable of synthesising and processing a variety of ECM. To date, it is unclear whether this expression of ECM by tumour cells only serves to produce the tumour stroma, or may also exert an effect on tumour cell differentiation and, possibly, tumour cell growth. Recent data on transmembrane connections between ECM and intracellular filaments suggesting a role for ECM in transducing signals for regulation of differentiation genes via cell membrane receptors (integrins) lend support to the hypothesis of the coexistence of a structural and a humoral signal pathway (Singer, 1979; Hay, 1983; Bissell *et al.*, 1982; Alberts *et al.*, 1989).

This work was supported by the Deutsche Forschungsgesellschaft (Lo 431 1/1). M.L. was the recipient of a Research Award of the Deutsche Gesellschaft für Innere Medizin. B.T. received a grant from the Bundesminister für Bildung und Wissenschaft.

We thank Ms G. Nowak for skilful technical assistance with the nude mice and Wolfram Ruf for reading the manuscript.

### References

ALBERTS, B., BRAY, D., LEWIS, J., RAFF, M., ROBERTS, K. & WATSON, J.D. (eds) (1989). The cytoskeleton. In *Molecular Biology of the Cell*, pp. 613–680. Garland: New York.

ALITALO, K., KEKSI-OJA, J. & VAHERI, A. (1981). Extracellular matrix proteins characterize human tumor cell lines. *Int. J. Cancer*, **27**, 755–761.

BARSKY, S.H., SIEGAL, G., JANOTTA, F. & LIOTTA, L.A. (1983). Loss of basement membrane components by invasive tumors but not by their benign counterparts. *Lab. Invest.*, **49**, 140–148.

BISSELL, M.J., HALL, H.G. & PARRY, G. (1982). How does the extracellular matrix direct gene expression? *J. Theor. Biol.*, **99**, 31–68.

BOWLES, N.E., RICHARDSON, P.J., OLSEN, E.G. & ARCHARD, L.C. (1986). Detection of Coxsackie B virus specific RNA sequences in myocardial biopsy samples from patients with myocarditis and dilated cardiomyopathy. *Lancet*, **i**, 1120–1122.

BÄTGE, B., BOSSLET, K., SEDLACEK, H.H., KERN, H.F. & KLÖPPEL, G. (1986). Monoclonal antibodies against CEA-related components discriminate between pancreatic duct type carcinomas and nonneoplastic duct lesions as well as non-duct type neoplasias. *Virch. Arch. A*, **408**, 361–374.

CHEN, Y.F., PAN, G.Z., HOU, X., LIU, T.H., CHEN, J., YANAIHARA, C. & YANAIHARA, N. (1990). Epidermal growth factor and its receptor in human pancreatic carcinoma. *Pancreas*, **5**, 278–283.

CHIRGWIN, J.M., PRZYBYLA, A.E., MACDONALD, R.J. & RUTTER, W.J. (1979). Isolation of biologically active ribonucleic acid from sources enriched in ribonuclease. *Biochemistry*, **18**, 5294–5299.

CLEUTJENS, J.P.M., HAVENITH, M.G., BEEK, C., VALLINGA, M., TEN KATE, J. & BOSMAN, F.T. (1990). Origin of basement membrane type IV collagen in xenografted human epithelial tumor cell lines. *Am. J. Pathol.*, **136**, 1165–1172.

DAMJANOV, I., DAMJANOV, N., KNOWLES, B.B. & ENGVALL, E. (1985). Origin of laminin in the extracellular matrix of human tumor xenografts in nude mice. *Virch. Arch. B*, **49**, 45–52.

FEINBERG, P. & VOGELSTEIN, B. (1983). A technique for radiolabeling DNA restriction endonuclease fragments to high specific activity. *Anal. Biochem.*, **132**, 6–13.

GIOVANELLA, B.C. & FOGH, J. (1985). The nude mouse in cancer research. *Adv. Cancer Res.*, **44**, 69–120.

HABERERN, C.L. & KUPCHIK, H.Z. (1985). Diversity of adhesion to basement components of human pancreatic adenocarcinomas. *Cancer Res.*, **45**, 5246–5251.

HABERERN, C.L. & KUPCHIK, H.Z. (1985). Diversity of adhesion to basement components of human pancreatic adenocarcinomas. *Cancer Res.*, **45**, 5246–5251.

HABERERN-BLOOD, C., LIOTTA, L.A., RAO, C.N. & KUPCHIK, H.Z. (1987). Laminin expression by human pancreatic carcinoma cells in the nude mouse and in culture. *J. Natl Cancer Inst.*, **79**, 891–898.

HAGLUND, C., ROBERTS, P.J., NORDLING, S. & EKBLOM, P. (1984). Expression of laminin in pancreatic neoplasms and in chronic pancreatitis. *Am. J. Surg. Pathol.*, **8**, 669–676.

HAGLUND, C., LINDGREN, P.J., ROBERTS, P.J. & NORDLING, S. (1986). Gastrointestinal cancer-associated antigen CA 19-9 in histological specimens of pancreatic tumours and pancreatitis. *Br. J. Cancer*, **53**, 189–195.

HAGLUND, C., ROBERTS, P.J. & NORDLING, S. (1989). Expression of laminin in benign and malignant sclerosing lesions of extrahepatic bile ducts. *J. Clin. Pathol.*, **42**, 927–930.

HALL, P.A., COATES, P.J., DEL BRUNO, R. & 4 others (1990). Patterns of cellular differentiation in human fetal, adult and pathological exocrine pancreas: correlation with in vitro and xenografted studies (abstract). *J. Pathol.*, **161**, 339a.

HAY, E.D. (1983). Cell and extracellular matrix: their organization and mutual dependence. *Mod. Cell Biol.*, **2**, 509–548.

HEINZE, H., ARNOLD, H.H., FISCHER, D. & KRUPPA, J. (1988). The primary structure of the human ribosomal protein S6 derived from a cloned cDNA. *J. Biol. Chem.*, **263**, 4139–4144.

HOOFF, A.V.D. (1983). Connective tissue changes in cancer. *Int. Rev. Connect. Tiss. Res.*, **10**, 395–432.

JOHNSON, G.D. & DE NOGUEIRA ARNAJO, G.M. (1981). A simple method of reducing the fading of immunofluorescence during microscopy. *J. Immunol. Methods*, **43**, 349–350.

JUST, M., HERBST, H., HUMMEL, M., DURKOP, H., TRIPIER, D., STEIN, H. & SCHUPPAN, D. (1991). Undulin is a novel member of the fibronectin tenascin family of extracellular matrix proteins. *J. Biol. Chem.*, **266**, 17326–17332.

KALLIONINEN, M., AUTIO-HARMAINEN, H., DAMMERT, K., RISTELI, J. & RISTELI, L. (1984). Discontinuity of the basement membrane in fibrosing basocellular carcinomas and basosquamous carcinomas of the skin: an immunohistochemical study with human laminin and type IV collagen antibodies. *J. Invest. Dermatol.*, **82**, 248–251.

KALTHOFF, H., ROEDER, C., HUMBURG, I., THIELE, H.G., GRETEN, H. & SCHMIEGEL, W. (1991). Modulation of platelet-derived growth factor A- and B-chain/c-sis mRNA by tumor necrosis factor and other agents in adenocarcinomas cells. *Oncogene*, **6**, 1015–1021.

KERN, H.F., RAUSCH, U. & MOLLENHAUER, J. (1986). Fine structure of human pancreatic adenocarcinoma. In *The Exocrine Pancreas. Biology, Pathobiology, and Diseases*, Go, V.L.W., Gardner, J.D., Brooks, F.P., Lebenthal, E., DiMagno, E.P. & Scheele, G.A., (eds) pp. 637–647. Raven Press: New York.

KERN, H.F., RÖHER, H.D., VON BÜLOW, M. & KLÖPPEL, G. (1987). Fine structure of three major grades of malignancy of human pancreatic adenocarcinoma. *Pancreas*, **2**, 2–13.

KLÖPPEL, G. (1989). Cancer of the pancreas: morphological and biological aspects. In *Cancer of the Bile Ducts and Pancreas*, Preece, P.E. & 2 others (eds) pp. 125–138. W.B. Saunders: Philadelphia.

KLÖPPEL, G. & FITZGERALD, P.J. (1986). Pathology of nonendocrine pancreatic tumors. In *The Exocrine Pancreas. Biology, Pathobiology, and Diseases*. Go, V.L.W., Gardner, J.D., Brooks, F.P., Lebenthal, E., DiMagno, E.P. & Scheele, G.A. (eds) pp. 649–674. Raven Press: New York.

KLÖPPEL, G., LINGENTHAL, G., BÜLOW, M.V. & KERN, H.F. (1985). Histological and fine structural features of pancreatic ductal adenocarcinomas in relation to growth and prognosis: studies in xenografted tumours and clinico-histopathological correlation in a series of 75 cases. *Histopathology*, **9**, 841–856.

KORC, M. (1991). Growth factors and pancreatic cancer. *Int. J. Pancreatol*, **9**, 87–91.

KYRIAZIS, A.P., KYRIAZIS, A.A., SCARPELLI, D.G., FOGH, J., RAO, M.S. & LEPERA, R. (1982). Human pancreatic adenocarcinoma line Capan-1 in tissue culture and the nude mouse. *Am. J. Pathol.*, **106**, 250–260.

LEMOINE, N.R. & HALL, P.A. (1990). Growth factors and oncogenes in pancreatic cancer. *Baillière's Clin. Gastroenterol.*, **4**, 815–832.

LIEHR, R.M., MELNYKOVYCH, G. & SOLOMON, T.E. (1990). Growth effects of regulatory peptides on human pancreatic cancer lines PANC-1 and MIA PaCa-2. *Gastroenterology*, **98**, 1666–1674.

LIOTTA, L.A., RAO, C.N. & BARKSY, S.H. (1984). Tumor cell interaction with the extracellular matrix. In *The Role of Extracellular Matrix in Development*, Liotta, L.A. (ed.) pp. 357–371. Alan R. Liss: New York.

LIPKIN, W.I., BATTENBERG, E.L.F., BLOOM, F.E. & OLDSTONE, M.B.A. (1988). Viral infection of neurons can depress neurotransmitter mRNA levels without histologic injury. *Brain Res.*, **451**, 333–339.

LÖHR, M., MÜLLER, M.K., GOEBELL, H. & KLÖPPEL, G. (1989). Prostaglandin analogue protects pancreatic B-cells against cyclosporin A toxicity. *Experientia*, **45**, 352–355.

LÖHR, M. & OLDSTONE, M.B.A. (1990). Detection of cytomegalovirus nucleic acid sequences in pancreas in type 2 diabetes. *Lancet*, **336**, 644–648.

LONGNECKER, D.S., JAMIESON, J.D. & ASCH, H.L. (1989). Regulation of growth and differentiation in pancreatic cancer (conference report). *Pancreas*, **4**, 256–275.

MAHLBACHER, V., SEWING, A., ELSÄSSER, H.P. & KERN, H.F. (1992). Hyaluran is a secretory product of human pancreatic adenocarcinoma cells. *Eur. J. Cell Biol.*, **58**, 28–34.

MAI, M., BRUNE, K., JACOBY, B., KERN, H.F. & MOLLENHAUER, J. (1990). Laminin interactions with ductal pancreatic adenocarcinoma cells: identification of laminin- and collagen-binding proteins. *J. Cell Sci.*, **95**, 65–74.

MÄKELA, J.K., RAASSINA, M. & VUORIO, E. (1988). Human pro$\alpha$1(I) collagen: cDNA sequence for the C-propeptide domain. *Nucl. Acid Res.*, **16**, 349.

MANIATIS, T., FRITSCH, E.F. & SAMBROOK, J. (1982). *Molecular Cloning. A Laboratory Manual.* Cold Spring Harbor Laboratory Press: Cold Spring Harbor, New York.

MOLLENHAUER, J., ROETHER, I. & KERN, H.F. (1987). Distribution of extracellular matrix proteins in pancreatic ductal adenocarcinoma and its influence on tumor cell proliferation in vitro. *Pancreas*, **2**, 14–24.

OLSEN, D., NAGAYOSHI, T., FAZIO, M., PELTONEN, J., JAAKKOLA, S., SANBORN, D., SASAKI, T., KUIVANIEMI, H., CHU, M.L., DEUTZMANN, R., TIMPL, R. & UITTO, J. (1989). Human laminin: cloning and sequence analysis of cDNAs encoding A, B1 and B2 chains, and expression of the corresponding genes in human skin and cultured cells. *Lab. Invest.*, **60**, 772–782.

PIHLAJANIEMI, T., TRYGGVASON, K., MYERS, J.C., KURKINEN, M., LEBO, R., CHEUNG, M.C., PROCKOP, D.J. & BOYD, C.D. (1985). cDNA clones coding for the pro-$\alpha$1(IV) chain of human type IV procollagen reveal an unusual homology of amino acid sequences in two halves of the carboxyl-terminal domain. *J. Biol. Chem.*, **260**, 7681–7687.

RAFIEE, P., HO, S.B., BRESALIER, R.S., BLOOM, E.J., KIM, J.H. & KIM, Y.S. (1992). Characterization of the cytokeratins of human colonic, pancreatic, and gastric adenocarcinoma cell lines. *Pancreas*, **7**, 123–131.

SANDBERG, M. & VUORIO, E. (1987). Localization of types I, II, and III collagen mRNAs in developing human skeletal tissue by in situ hybridization. *J. Cell Biol.*, **104**, 1077–1084.

SCHWARZBAUER, J.E., TAMKUN, J.W., LEMISCHKA, I.R. & HYNES, R.O. (1983). Three different fibronectin mRNAs arise by alternative splicing within the coding region. *Cell*, **35**, 421–428.

SEIFERT, D., KEETON, M., EGUCHI, Y., SAWDEY, M. & LOSKUTOFF, D.J. (1991). Detection of vitronectin mRNA in tissues and cells of the mouse. *Proc. Natl Acad. Sci. USA*, **88**, 9402–9406.

SINGER, I.I. (1979). The fibronexus: a transmembrane association of fibronectin-containing fibers and bundles of 5 nm microfilaments in hamster and human fibroblasts. *Cell*, **16**, 675–685.

TRAUTMANN, B., SCHLITT, H.J., HAHN, E.G. & LÖHR, M. (1993). Isolation, culture, and characterization of human pancreatic duct cells. *Pancreas*, **8**, 248–254.

USCANGA, L., KENNEDY, R.H., STOCKER, S., GRIMAUD, J.-A. & SARLES, H. (1984). Immunolocalisation of collagen types, laminin and fibronectin in the normal human pancreas. *Digestion*, **30**, 158–164.

ZETTER, B.R. (1990). The cellular basis of site-specific tumor metastasis. *N. Engl J. Med.*, **322**, 605–612.